# Adaptation strategies of horses with induced forelimb lameness walking on a treadmill

**DOI:** 10.1111/evj.13344

**Published:** 2020-09-24

**Authors:** Filipe M. Serra Bragança, Elin Hernlund, Maj H. Thomsen, Nina M. Waldern, Marie Rhodin, Anna Byström, P. René van Weeren, Michael A. Weishaupt

**Affiliations:** ^1^ Department of Equine Sciences Faculty of Veterinary Medicine Utrecht University Utrecht The Netherlands; ^2^ Department of Anatomy, Physiology and Biochemistry Swedish University of Agricultural Sciences Uppsala Sweden; ^3^ Department of Veterinary Clinical Sciences Faculty of Health Sciences University of Copenhagen Taastrup Denmark; ^4^ Equine Department Vetsuisse Faculty University of Zurich Zurich Switzerland

**Keywords:** horse, kinetics, kinematics, compensatory mechanisms, walk, trot

## Abstract

**Background:**

There is a paucity of research describing the gait pattern of lame horses at the walk.

**Objectives:**

To describe the changes in motion pattern and vertical ground reaction forces (GRFz) in horses with induced forelimb lameness at the walk and compare those changes with the changes observed at the trot.

**Study design:**

Experimental study.

**Methods:**

In 10 clinically sound Warmblood horses, moderate forelimb lameness was induced using a sole pressure model followed by trot and walk on a treadmill. Kinematic data were collected using 3D optical motion capture (OMC), and GRFz by an instrumented treadmill. Mixed models were used to compare sound baseline versus forelimb lameness (significance was set at *P* < .05).

**Results:**

Lameness induction significantly reduced peak GRFz on the second force peak, and vertical impulse in the lame limb. Stride and stance duration in all limbs were reduced. Lameness significantly affected the vertical movement symmetry of the head and withers. Maximum limb retraction angle, fetlock extension and protraction speed were reduced in the lame limb. Body centre of mass (COM) translation was reduced in the side‐to‐side direction and increased in the vertical and fore‐aft directions. Several compensatory kinetic and kinematic changes were observed in the nonlame limbs. The observed changes in both kinetics and kinematics were generally smaller at walk with fewer variables being affected, compared to the trot.

**Main limitations:**

Only one degree and type of orthopaedic pain (sole pressure) was studied.

**Conclusions:**

Compensatory strategies of forelimb lameness at the walk include alteration of several kinetic and kinematic parameters and have some specific patterns and inter‐individual differences that are not seen at the trot. However, much like at the trot, head movement and forelimb vertical force symmetry seem to be the most useful parameters to detect forelimb lameness at walk.

## 1 INTRODUCTION

Movement symmetry parameters at the trot are the main tools for subjective and objective gait assessment in the horse. Several kinetic and kinematic parameters have been validated as clinically relevant lameness indicators.^1^ These include vertical ground reaction forces^2^ and symmetries of the vertical excursions of the head, withers and pelvis,^3^ which reflect compensatory mechanisms generally related to load redistribution.^4^, ^5^, ^6^

At the walk, compensatory strategies for lameness are not well understood and no specific gait parameters have yet been proposed for clinical use. However, lameness evaluation at the walk can be essential in horses with conditions prohibiting the support of loads associated with trot, for example, in the immediate post‐surgical period. A head and neck nod has been described as a visual indicator of forelimb lameness in horses. Also, some locomotor disorders due to mechanical or neurological pathology may manifest more obviously at walk.^7^ It has even been suggested that gait analysis at the walk may be more sensitive in detection of subtle lameness than trot.^8^ Despite this, kinematic and kinetic variables suitable for objective lameness detection at walk have received little attention. One study on single supporting limb lameness at walk identified decreased vertical ground reaction force (GRFz) in the lame limb, compensated by an increased GRFz in the remaining limbs. Also a reduction of the horizontal craniocaudal ground reaction force (GRFy) in the lame limb with increased GRFy in the contralateral forelimb and ipsilateral hindlimb was seen.^9^ In another experiment, stride and stance duration, joint angles and head movement adaptations changed in a similar way in both walk and trot, but to a lesser extent at the walk. Vertical movement symmetry indices of the withers and croup were deemed not useful as lameness indicators for walk, as the changes were too small.^3^

While the trot is a diagonal gait with a suspension phase, the walk is a four‐beat, symmetric gait without suspension. At the walk, diagonal and ipsilateral bipedal support alternate with tripedal support phases. The withers and croup are raised and lowered out of phase, with the highest position of the withers occurring at mid stance of each forelimb and for the croup at mid stance of each hindlimb.^3^ The head is raised and lowered out of phase with the withers and in phase with the croup, which is seen as an energy‐conserving mechanism.^10^ The vertical ground reaction force curve has a double‐peak shape with the dip coinciding with fore‐/hindlimb midstance.^11^ This pattern is best explained by a spring‐mass model with overlaps of contralateral limb support.^12^

Because of the differences in gait mechanics between walk and trot, it can be presumed that lameness has a different influence on the two gaits. The aim of this study was to gain a better insight into the changes in motion pattern and vertical ground reaction forces in horses with a moderate degree of induced forelimb lameness at the walk. We specifically aimed to identify kinematic and kinetic variables with a reasonable sensitivity and specificity to discriminate between lame and nonlame measurements that could be further explored for objective assessment at the walk in clinical lameness cases.

## 2 MATERIALS AND METHODS

### 2.1 Horses

The dataset used for the study was collected during experimental sessions described previously.^13^ Briefly, 10 horses were included in this study and all were considered clinically sound when examined by a veterinarian surgeon experienced in orthopaedic exams (M.A.W.). All were Warmblood geldings with an age range of 5‐21 years and a mean height at the withers of 169 ± 6.3 cm (range 161‐180 cm). Horses were trained regularly and used in jumping and/or dressage competitions at amateur level. Prior to the experiment, horses were acclimatised to the treadmill and experimental setup.^14^

### 2.2 Lameness induction

Each horse was shod with modified horseshoes with M10 nuts welded to the inner rim of each branch.^15^ Before nailing on the shoes, the soles were cleaned and trimmed to a consistent thickness. Lameness was induced by screwing bolts with flat tips into the nuts, thereby applying pressure to induce a nociceptive stimulus to the corium of the sole (Figure S1). The procedure was controlled using a torque meter with 0.1 Nm increments (Type 757, Rahsol Dremotec, Gedore Group, Remscheid, Germany), to ensure that the same torque was applied to the medial and lateral half of the hoof. The goal was to induce different degrees of reversible supporting lameness in each horse, evaluated subjectively by two experienced clinicians (M.H.T. and M.A.W.) using the following convention:

- degree 1/5 (subtle lameness), irregularity not visible on every stride at the trot.
- degree 2/5 (mild lameness), visible on every stride at the trot.
- degree 3/5 (moderate lameness), distinctly visible on every stride at the trot but without obvious disturbance to the cadence of movement.

### 2.3 Data collection

Kinetic data were collected with an instrumented treadmill (Mustang 2200, Kagra AG, Fahrwangen, Switzerland)^16^; kinematic data were recorded with 10 infrared 3D optical motion capture (OMC) cameras (Oqus 300+, Qualisys AB, Motion Capture Systems, 411 05, Göteborg, Sweden) that registered the positions of 52 skin‐mounted spherical reflective markers. For detailed marker placement, see Figure S2. Both measurement systems were synchronised in time using hardware‐based synchronisation. Sampling frequency was set at 512 Hz for force and 256 Hz for OMC data. Data collection lasted 20 seconds for each trial. Measurements were taken at the horse's preferred speed, which was set based on visual assessment of locomotion regularity.

Before lameness induction, each horse was subjected to a baseline measurement at walk and trot. Subsequently, the three lameness degrees were induced successively and measured at the same gait speed as the baseline measurements. The trot measurement with the highest lameness degree was immediately followed by a measurement at walk. Left and right limb lameness were induced on the same day in random order; the lameness induction trial in the contralateral limb took place as soon as kinetic data had returned to baseline values.

### 2.4 Data processing

The ground reaction force (GRF) exerted by each limb during stance was calculated by the treadmill software (HP2, University of Zurich) as previously described.^16^ During the measurements, the three‐dimensional coordinates of each marker were automatically tracked by the motion capture software (QTM, version 2.9, Qualisys AB, Motion Capture Systems, 411 05, Göteborg, Sweden). Complete datasets were exported to Matlab 2018b (MathWorks, Natick, Massachusetts, USA) for further analysis using custom‐written scripts. Force parameters were normalised to horse body mass. Stride segmentation was performed using the hoof‐on moments of the left forelimb. The beginning and end of stance phase of each limb was determined by the intersection of the linear approximation to the initial and terminal slope of the force curve with the zero‐baseline.^16^ The inbuilt speedometer registered the treadmill belt speed.

An overview of the measured or calculated temporal, kinetic and kinematic variables is presented in Table S1. Variables included in the analysis were selected based on previous publications investigating induced lameness at walk and trot.^1^, ^2^, ^3^, ^4^, ^5^, ^17^, ^18^, ^19^

### 2.5 Selection of left or right measurement trial

To ensure that the most relevant trials were submitted to statistical analysis, one trot trial per horse and the corresponding walk trial, either the left front induction or the right front induction, was selected based on (1) the larger difference in minimum head position between left and right stride half‐cycle (MinDiff), and (2) the larger difference in peak vertical forces (Fz_peak_) between front limbs measured at trot, since these two parameters have been shown to be well correlated to forelimb lameness.^1^

For the purposes of this manuscript, only the baseline data and the data of the highest degree of induced forelimb lameness at walk and trot (3/5) of each horse were used. Symmetry parameters from right forelimb lameness were multiplied by −1 to mirror the indices and thus categorise all data as if they were derived from left limb inductions only, reported as results of the ‘lame’ (left front) limb.

### 2.6 Dynamics of the centre of force at walk

In order to better understand the compensatory load redistribution between limbs in weightbearing lameness at walk, the centre of force (COF) relative to the horse's centre of mass (COM) was calculated. The approximate position of the COM was calculated based on the method previously described by Buchner et al.^20^ using solely the 3D coordinates of the markers located at the sternum and the dorsal spinal process of the lumbar vertebra L3.^21^ For calculating COF, both force and OMC data were used, and OMC data were resampled to match the frame rate of the force data. For each frame, the relative COF was calculated as:

$${\text{COF}}_{x,y}=\frac{\left({\text{LF}}_{\text{Fz}}*{\text{LF}}_{x,y}\right)+\left({\text{RF}}_{\text{Fz}}*{\text{RF}}_{x,y}\right)+\left({\text{LH}}_{\text{Fz}}*{\text{LH}}_{x,y}\right)+\left({\text{RH}}_{\text{Fz}}*{\text{RH}}_{x,y}\right)}{{\text{LF}}_{\text{Fz}}+{\text{RF}}_{\text{Fz}}+{\text{LH}}_{\text{Fz}}+{\text{RH}}_{\text{Fz}}}$$

where LF is the left front limb, RF is the right front limb, LH is the left hindlimb, RH is the right hindlimb, Fz is the vertical ground reaction force and (x, y) is the hoof position in the horizontal plane, relative to the treadmill coordinate system.

### 2.7 Data analysis

In order to identify the temporal, kinetic and kinematic variables that on a group level would be likely to be associated with lameness at the walk, sensitivity and specificity of the variables listed in Table S1 were calculated on a horse level using the Youden's index to determine the optimal sensitivity and specificity cut‐off value. From each horse and gait, sensitivity and specificity were calculated based on the difference between baseline and induction conditions. Thereafter, parameters with high sensitivity and specificity on a group level were investigated by averaging (using the group median) the sensitivity and specificity of all horses. This approach avoided investigating parameters with a very high inter‐individual variation and also allowed to explore the individual pattern for movement adaptation and load redistribution strategy of each horse.

**Figure 1:**
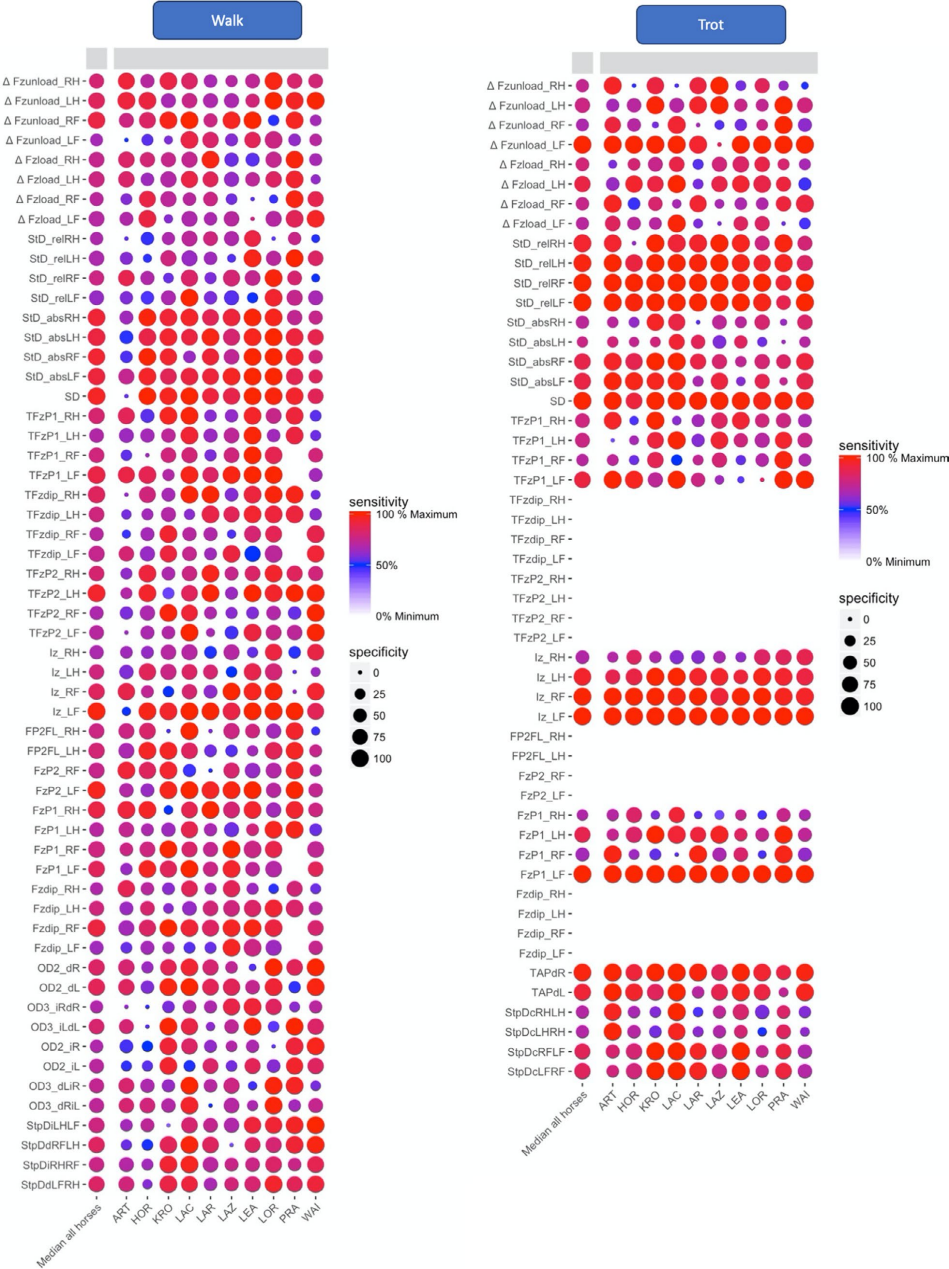
Heat map for the kinetic variables at walk (left) and trot (right). Each variable is located on the y‐axis and each horse on the x‐axis. Sensitivity is described by the colour pallet and specificity by the size of each dot. For variable names, please refer to Table S1

**Figure 2:**
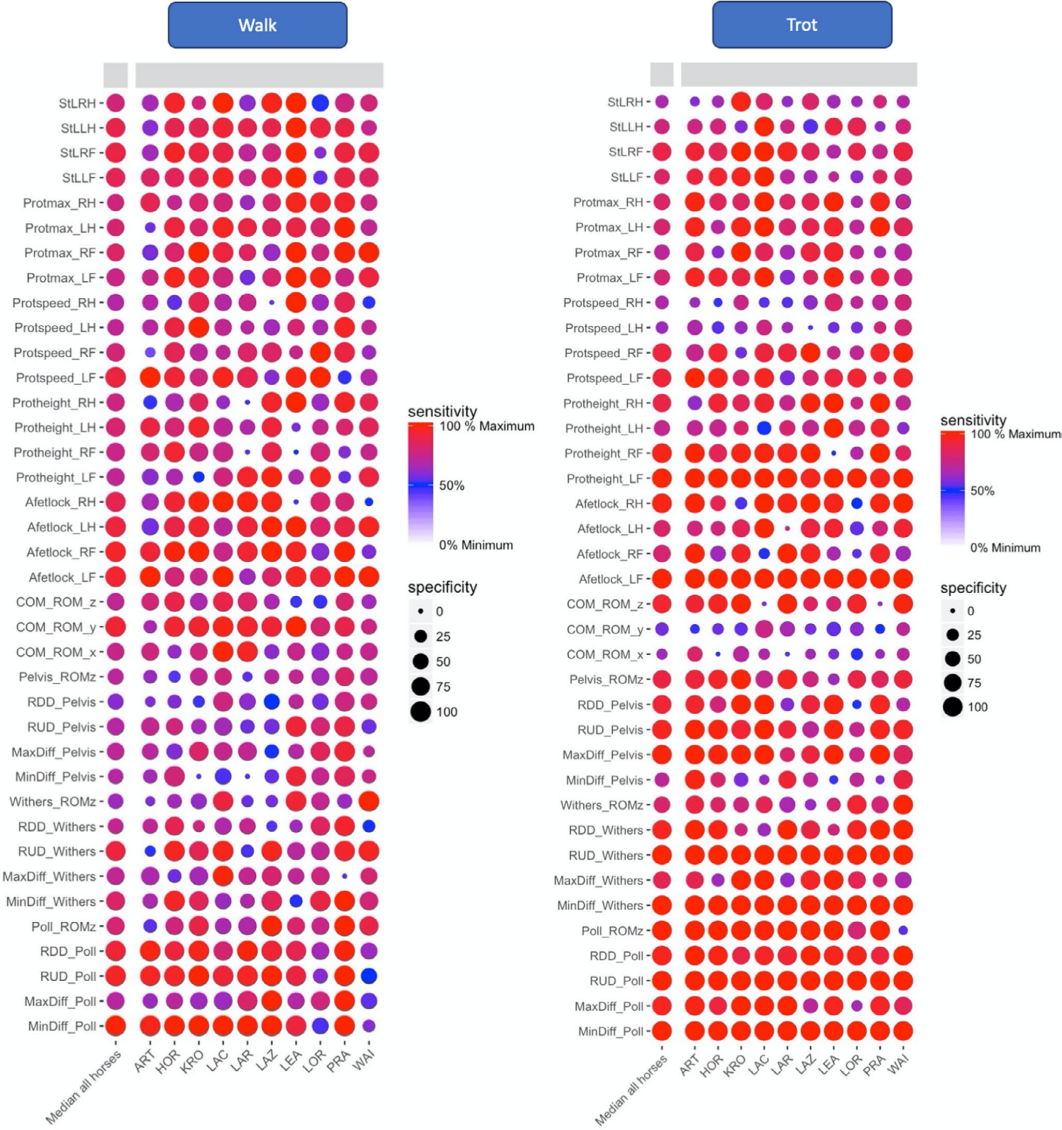
Heat map for the kinematic variables at walk (left) and trot (right). Each variable is located on the y‐axis and each horse on the x‐axis. Sensitivity is described by the colour pallet and specificity by the size of each dot. For variable names, please refer to Table S1

In order to compare sensitivities and specificities between walk and trot, variables were preferentially selected which could be calculated for both gaits. The sensitivity and specificity were calculated in R‐studio (R‐Studio, Boston, Massachusetts, USA) with the package pROC (version 1.10.0) using all collected strides from each horse, for walk and trot separately. The results were plotted in a heat map (Figures 1 and 2) for visual review. The parameters with the highest sensitivity and specificity on a group level were selected for further modelling.

Linear mixed models were created with each variable selected based on the sensitivity and specificity analysis as outcome. Stride level data for these variables were entered into the model from the baseline measurements and the induced lameness measurement at walk and trot from each horse. The models were calculated in R‐studio^5^ using the package lme4 (version 1.1‐15). In each model, horse was used as a random effect and the lameness condition (not lame = baseline and lame = induction) as fixed effect. Walk and trot were analysed in separate models. Model fit was evaluated using q‐q plots and box‐plots of the residuals. Model estimates were represented as least square means. P‐values were adjusted for multiple comparison using the Bonferroni correction.

## 3 RESULTS

The average walking speed was 1.68 m/s (range 1.57‐1.79 m/s) and the average trotting speed was 3.87 m/s (range 3.78‐3.94 m/s). For the walk, an average of 14.6 strides per trial (range 11‐17 strides) and for the trot trials, an average of 24 strides per trial (range 15‐29 strides) were used for statistical analysis. One lameness induction from one horse was lost due to a technical problem. For this subject, the only available induction (right front limb) and baseline measurements were used in the analysis. For the trials used in the statistical analysis, the mean (standard deviation) lameness score at the trot was 2.7 (0.4).

### 3.1 Temporal parameters

The effect of induced lameness at walk and trot on temporal parameters is summarised in Table S2. Significant changes in stride temporal parameters at walk included a reduction of stride duration (SD; −2.05%), reduction of absolute stance duration (StD_abs_) in all four limbs (more pronounced in the front limbs), and a delay of the occurrence of the first force peak (+8.4%) in the lame forelimb and in both hindlimbs. Other variables were also observed to change on an individual level (Figure 1), reflecting a more individual pattern of compensation. SD was shortened at both walk and trot with induced lameness. In contrast to the walk, StD_abs_ at the trot increased, in the lame and contralateral front limb. Relative stance duration (duty factor, StD_rel_) increased only slightly by +0.4% at the walk on the lame limb and ipsilateral hindlimb while at the trot, StD_rel_ had a more pronounced increase on all four limbs, mainly on both front limbs (+5.5%).

The inter‐limb step duration at walk was mainly affected by the decreased duration between ipsilateral hindlimb and lame front limb impacts, and increased duration between diagonal hindlimb and contralateral front limb impacts (see StpDi in Table S2). At trot, the main observed change in inter‐limb timing was a reduction of the step duration between the lame limb and contralateral front limb (StpDc). The time dissociation between hoof contacts of the diagonal limbs (TAP) was affected for both diagonals with the horses landing relatively earlier with the front limbs after lameness induction.

At the walk, the most obvious changes with regard to the duration of the bipedal and tripedal limb support phases involved the shortening of both diagonal two‐limb support phases and the prolongation of the three‐limb support phase, which included the lame forelimb (Table S2).

### 3.2 Force parameters

**Figure 3:**
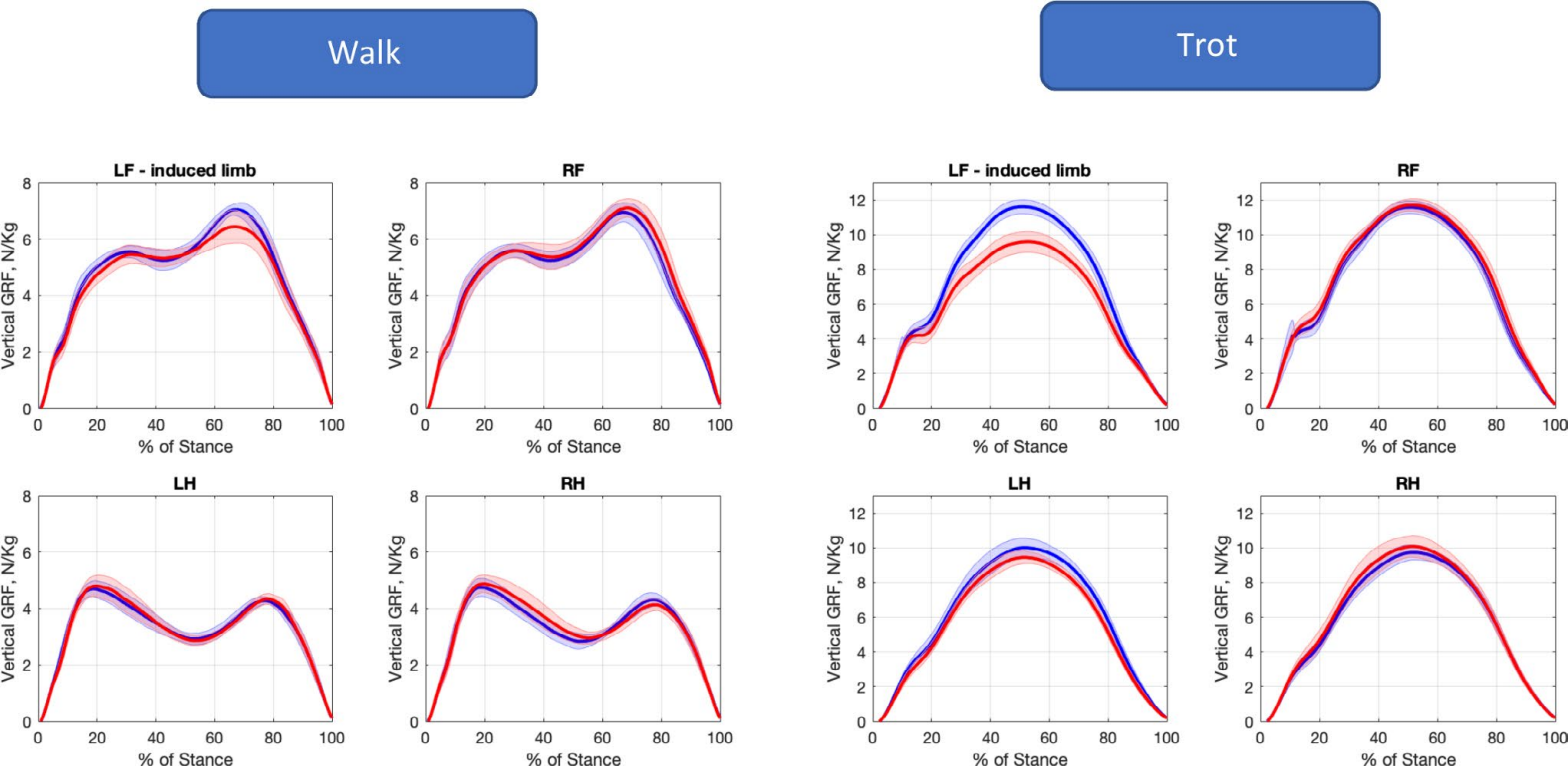
Vertical ground reaction forces of all four limbs at walk (left) and trot (right) before (blue) and after (red) lameness induction. Thick line represents the mean, and the shaded area represents the standard deviation of all horses. LF, left forelimb; RF, right forelimb; LH, left hindlimb; RH, right hindlimb

The effect of induced lameness at walk and trot on kinetic parameters is summarised in Table S2. At the walk, the primary effect was observed in the lame limb (Figure 3): vertical impulse (Iz) was reduced by −5.1%, vertical force of the first peak (Fz_peak1_) by −1.9% and vertical force of the second peak (Fz_peak2_) by −6.1%. In the diagonal hindlimb, Fz_peak1_ increased by +4.8%. In the contralateral forelimb, the loading rate (∆Fz_load_) increased by +16.5% and the unloading rate (∆Fz_unload_) by +15%.

In general, the changes were similar but more pronounced at trot (Iz −14.3%, Fz_peak_ −17.7% of the lame limb) than at the walk. The ∆Fz_load_ and ∆Fz_unload_ appeared to be more affected in the lame limb at the trot while at walk, the differences were occurring mainly in the contralateral front limb.

### 3.3 Kinematic parameters

**Figure 4:**
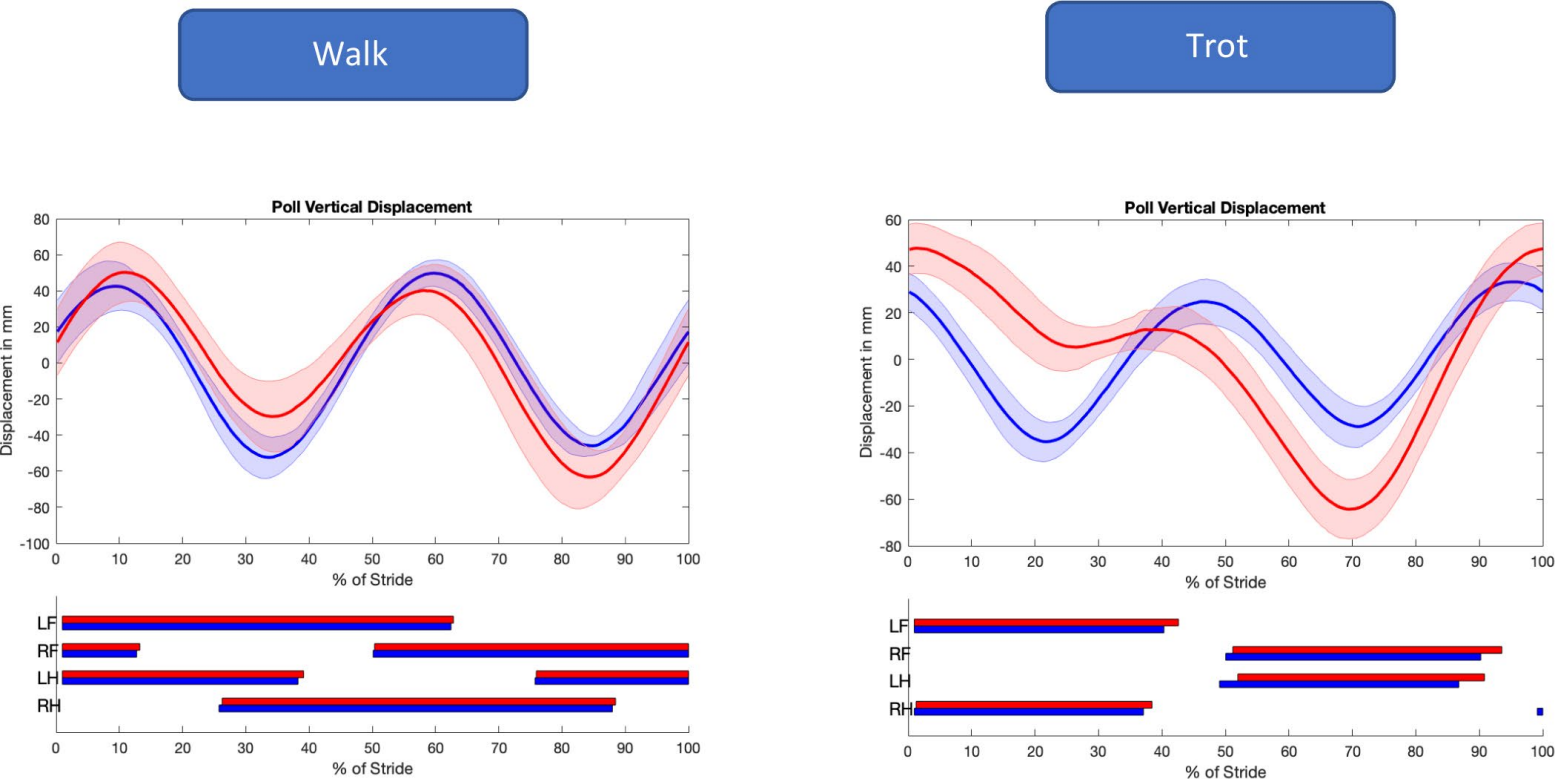
Vertical displacement of the poll marker at walk (left) and trot (right) before (blue) and after (red) lameness induction. Thick line represents the mean, and the shaded area represents the standard deviation of all horses. Below, stance phase of the four limbs is presented in blue before and red after lameness induction. LF, left forelimb; RF, right forelimb; LH, left hindlimb; RH, right hindlimb

The effects of induced lameness at walk and trot on kinematic parameters are summarised in Table S3. At the walk, the most prominent changes in upper body kinematics were observed for the MinDiff and in the Range Up Difference (RUD) and Range Down Difference (RDD) of the vertical displacement of the head (poll) (Figure 4). The vertical displacement of the withers was affected but to a much smaller magnitude. The range of motion of the vertical displacement (ROM_z_) of the head was only affected at the trot and increased by +50.5% after lameness induction.

**Figure 5:**
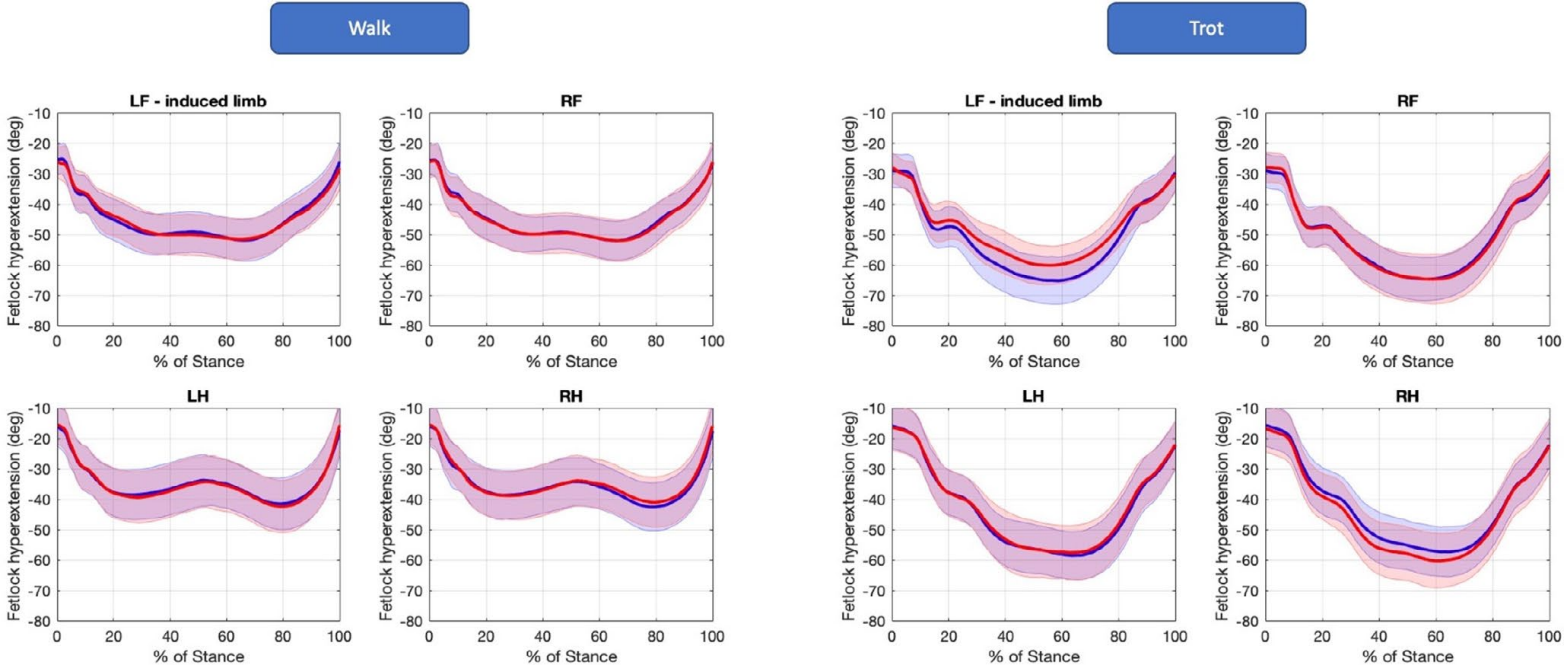
Fetlock hyperextension angle of all four limbs during stance phase at walk (left) and trot (right), before (blue) and after (red) lameness induction. Thick line represents the mean, and the shaded area represents the standard deviation of all horses. LF, left forelimb; RF, right forelimb; LH, left hindlimb; RH, right hindlimb

Regarding limb kinematics at walk, both maximal protraction and retraction angles were reduced, except maximal protraction of the lame limb and maximal retraction of the ipsilateral hindlimb (LH). Maximal fetlock hyperextension (Figure 5) was reduced in the lame limb (−3.3%) and increased in the other limbs. Maximal limb speed during the swing phase was also reduced in the lame limb (−4.6%) and increased in both hindlimbs (+2.4% and +2.7% respectively). As for StD_abs_, stance length (StL) was reduced in all four limbs. Other variables were also observed to change on an individual level (Figure 2), reflecting a more individual pattern of compensation.

Maximal protraction height at the walk only increased in the contralateral front limb (2.5%) and at the trot, the most pronounced change was a decrease in the lame limb after lameness induction (−16.8%). Maximal protraction was increased in the lame limb (+1.6%) at the trot, in contrast to the walk where maximal protraction remained unaffected. Maximal retraction of the lame limb was reduced for both walk and trot (−2.6% and −2.8% respectively).

### 3.4 COM translations

**Figure 6:**
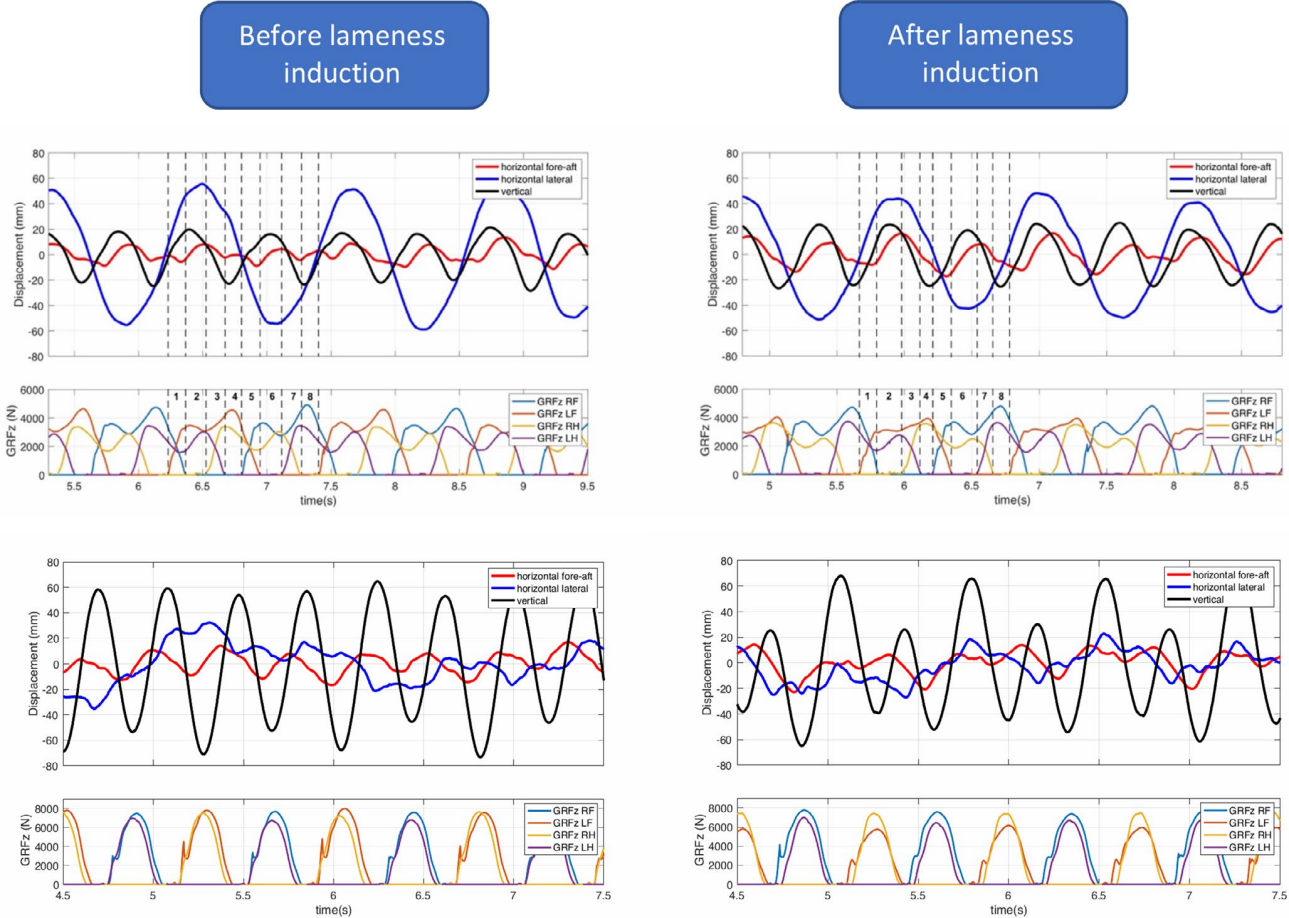
Representative translation movements of the body centre of mass (COM) of one horse. Walk before (top left) and after (top right) lameness induction. Trot before (bottom left) and after (bottom right) lameness induction. Note that at the walk, the greatest component of translation is the horizontal lateral translation while at the trot, it is the vertical translation. Also, note the asymmetry of the maximum vertical (black) translation at the trot, between the two diagonals after lameness induction. 1: tripedal support (RF, LH, LF); 2: left ipsilateral bipedal support (LH, LF); 3: tripedal support (LH, LF, RH); 4: left diagonal bipedal support (LF, RH); 5: tripedal support (LF, RH, RF); 6: right ipsilateral bipedal support (RH, RF); 7: tripedal support (RH, RF, LH); 8: right diagonal bipedal support (RF, LH)

At walk, the main translation of the body COM relative to the treadmill was in the latero‐lateral direction (Figure 6). This was significantly reduced after lameness induction with an increase of the remaining translations (horizontal fore‐aft and dorsoventral). At the trot, the main translation of the COM was in the vertical (dorsoventral) direction and this was significantly reduced after lameness induction.

### 3.5 COM‐COF difference

**Figure 7:**
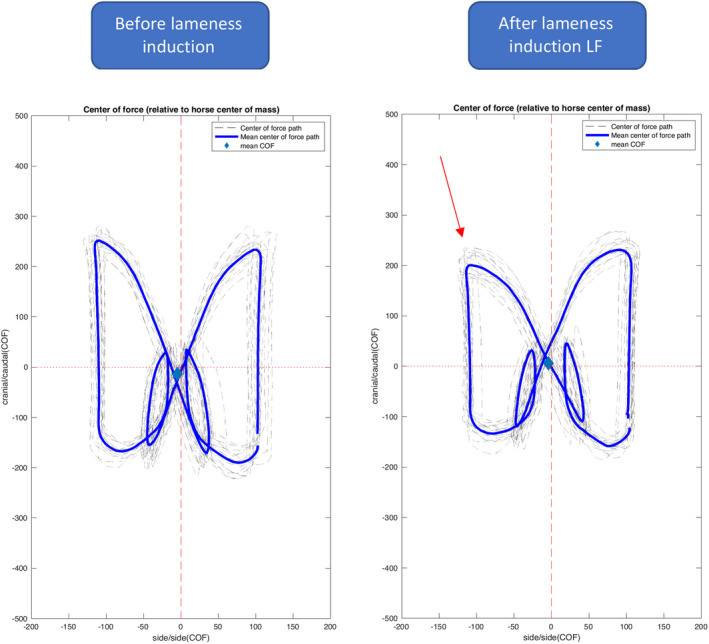
Representative centre of force (COF) path relative to centre of mass (COM), before (left) and after (right) left front lameness induction of one horse in mm. On the x‐axis, left (negative) and right (positive) relative to the COM. On the y‐axis, cranial (positive) and caudal (negative) relative to the horse COM. Red arrow indicates the area of caudal displacement of the COF over the lame left front limb

The general pattern of the COF relative to COM path at walk was very characteristic (butterfly‐like) and similar between horses (Figure 7, Videos S1 and S2). Based on visual inspection of these figures, after lameness induction at the walk, horses reduced the maximal cranial translation of the COF, relative to the COM, towards the lame limb (Figure 7). This was observed in 70% of the selected lameness inductions.

## 4 DISCUSSION

Understanding the compensatory mechanisms of adaptation to lameness and the different strategies employed at different gaits is essential to evaluate lameness in horses. The compensatory mechanisms are complex and demonstrate that in general, kinetic and kinematics of the nonlame limbs are also affected by lameness induction in a single limb (here front limb), making visual lameness assessment challenging. There seems to be different compensatory strategies employed for the different gaits; parameters with known relation to lameness at trot might not be applicable to lameness at the walk.

We believe that our approach for identifying useful variables on an individual basis, using heat maps, allowed us to better understand individual variation in adaptation to lameness. The heat maps showed that there was indeed individual differences in strategies for adapting to sole pressure‐induced lameness. They further showed that these inter‐individual differences in compensatory strategies were less pronounced at trot than at walk, for example, for vertical impulse (Iz) (Figures 1 and 2). We hypothesise that this is related to the fact that walk with its bipedal and tripedal support phases increases the possible strategies to compensate.

The lameness‐induced changes in both kinetics and kinematics were much smaller at walk than at trot. This has been previously described in other studies in the walk after lameness induction,^3^, ^17^ and is the reason why the trot is the preferred gait to visually assess lameness in a clinical setting as described by Ross et al.^22^ The phenomenon can possibly be explained by the fact that horses are moving at higher speed and stride frequency, only have bipedal support and the COM translation is mainly happening in the vertical direction; this results in the higher Fz_peak_ observed at trot compared to the walk (Table S2). Ultimately, this will result in higher levels of pain/discomfort experienced by the horse during the stance phase of the lame limb at the trot.

### 4.1 Kinetics

The changes in limb loading observed in this study at the trot were in agreement with previous publications,^5^, ^18^, ^23^, ^24^, ^25^, ^26^, ^27^ with the reduction of Fz_peak_ and Iz in the lame limb as most significant changes (Figure 3). Interestingly, at both gaits, only a very mild increase in contralateral front limb Fz_peak_ was observed compared to the reduction in the lame limb (Figure 4). Previous publications have reported no increased loading with regard to Fz_peak_ of the contralateral limb in front limb^5^ and hindlimb^4^ induced lameness at trot. This finding was interpreted as the result of the prolonged StD_abs_ seen in the lame as well as in the contralateral limb.^4^, ^5^, ^28^

When looking at vertical impulse (Iz), there was an increase in the contralateral front limb, as previously described for hindlimb lameness,^4^ and reduction in the ipsilateral hindlimb at the trot. At the walk, Iz was reduced in the lame limb and in the ipsilateral hindlimb without significant increases in contralateral limbs. This is likely related to the observed reduction in SD and StD_abs_ of all four limbs, which is in contrast to the trot where front limb StD_abs_ increases after lameness induction.

Changes in limb loading at walk as a result of lameness were previously reported in a study using stationary force plates. That study showed a reduction of both force peaks and a flattening of the force dip in the lame limb.^18^ We can confirm these observations, as we have found a reduction of both force peaks (predominantly of forelimb Fz_peak2_), although we have failed to find an increase/flattening of the force dip in the lame limb. Furthermore, Fz_peak1_ was increased in the diagonal hindlimb and contralateral front limb (incl. Fz_peak2_), which reflects the mechanism of force redistribution from the lame limb to the following limbs in the step sequence.^29^

The inter‐limb timing at walk was mainly affected by the step duration from the ipsilateral hindlimb to the lame limb, indicating an attempt to place the lame forelimb relatively earlier in the stride cycle. This compensatory mechanism has been previously reported.^28^ In combination with the subsequent prolonged tripedal support phases, the horse tries to distribute the load away from the affected limb to the diagonal hindlimb and contralateral forelimb. Furthermore, the shortened diagonal bipedal support phases indicate that one of the main strategies of lameness adaptation at the walk is to reduce the lateral oscillation of the COM (Figure 6).

The time dissociation between diagonal limbs (TAP) at the trot was affected for both diagonals with the horses landing earlier with the respective front limbs after lameness induction (Table S2), confirming previous observations.^5^ This is likely due to the increased StD_abs_ of the front limbs after lameness induction.

### 4.2 Kinematics

Changes in upper body kinematics were more obvious at trot than at walk. Total range of motion of the vertical displacement of the head (ROMz) was increased by 50% after lameness induction at trot, but there was no change seen at walk. Still, based on our heat map analysis and the significant differences between lame and sound measurements, motion symmetry of the poll was one of the most sensitive and specific parameters on a group level, supporting previous publications that suggest that head motion symmetry can be of significant importance at trot and at walk.^3^, ^8^ In our study, one horse (Figures 1 and 2, LAZ) had a significant baseline asymmetry of the vertical displacement of the poll, yet the horse showed no weightbearing asymmetry on the GRFz of the front limbs and the poll asymmetry was not present at the trot. This indicates that a head nod at the walk could render false positive results if used for detection of weightbearing lameness. Therefore, the sensitivity and specificity of the poll motion asymmetry for lameness detection at walk need to be determined in a larger population of horses.

Withers motion symmetry was also affected, but in a smaller magnitude compared to the head, as described previously.^3^, ^30^ Asymmetry of the vertical displacement of the withers has been related to maximal protraction and retraction angle of the forelimbs and might not be entirely due to lameness, as it can be related to motor laterality.^31^ Therefore, motion symmetry of the withers at the walk should be interpreted with caution in the light of lameness diagnosis.

The reduced maximal cranial displacement of the COM‐COF difference towards the lame limb occurred at the moment of transition between tripedal support and the following ipsilateral bipedal support phase (see Videos S1 and S2). This occurred just prior to Fz_peak1_ of the forelimb and the moment of force dip of the ipsilateral hindlimb. We hypothesise that the reduction of the most cranial displacement of COF towards the lame limb, in comparison to the contralateral front limb (Figure 7), is the explanation for the reduced Iz and force peaks of the lame limb. We believe that this mechanism is an attempt to bring the COF closer to the COM, reducing loading of the lame limb. This is likely also coupled to the reduced latero‐lateral translation of the body COM after lameness induction. Further research is needed to better describe and investigate this COF path and the observed changes in body COM.

## 5 LIMITATIONS OF THE STUDY

Horses were investigated on a treadmill at a constant speed; it may therefore not be appropriate to extrapolate the results to over ground locomotion. Further studies are needed to assess the validity of the variables used in this study over ground, where a higher inter‐stride variation is expected that will ultimately affect the sensitivity and specificity of some of the variables presented here. There is also a need to confirm these results in horses with naturally occurring lameness caused by different orthopaedic pathologies and with pain arising from different anatomical locations in the limb.

## 6 CONCLUSIONS

Marked differences exist between the compensatory mechanisms of adaptation to lameness between walk and trot. Specifically, we suggest that the kinetic variables Iz, Fz_peak1_, Fz_peak2_ and StpDi are interesting candidate parameters that can be used for objective lameness assessment at the walk. For the kinematics, we suggest that the symmetry variables for the head MinDiff, RUD, RDD, Prot_speed_ and A_fetlock_ are also interesting candidates. Nevertheless, some of the described parameters may not be easily perceived in a clinical situation and are possibly only measurable on a treadmill. Based on current IMU and OMC technologies available, the head symmetry parameters are likely good candidates for objective forelimb lameness assessment at walk in a clinical setting.

## ETHICAL ANIMAL RESEARCH

The experimental protocol was approved by the Animal Health and Welfare Commission of the canton of Zürich (permission number 51/2013).

## OWNER INFORMED CONSENT

Informed consent for data collection was obtained from the horse owners prior to the study.

## CONFLICT OF INTERESTS

No competing interests have been declared.

## AUTHOR CONTRIBUTIONS

M.A. Weishaupt, M. Rhodin, M.H. Thomsen, N.M. Waldern and E. Hernlund designed and executed the study. F.M. Serra Bragança, M.A. Weishaupt and E. Hernlund analysed the data, interpreted the results and prepared the manuscript. All authors revised the manuscript critically and gave the final approval of the manuscript.

### PEER REVIEW

The peer review history for this article is available at https://publons.com/publon/10.1111/evj.13344.

## Supporting information

Fig S1

Fig S2

Table S1

Table S2

Table S3

Video S1

Video S2

## Data Availability

The data that support the findings of this study are available from the corresponding author upon reasonable request.
