# Supplementary material for: Adaptation strategies of horses with induced forelimb lameness walking on a treadmill
Source: Equine Vet J. 2020 Sep 24;53(3):600–11. doi: 10.1111/evj.13344 (PMC8048804; doi:10.1111/evj.13344)

Figure S1: One of the study subjects, equipped with a modified shoe. Please note the 2 M10 nuts welded to the inner rim of each branch. Lameness was induced by screwing bolts with flat tips into the nuts. In order to ensure that the same torque was applied to the medial and lateral half of the hoof a torque meter was used.

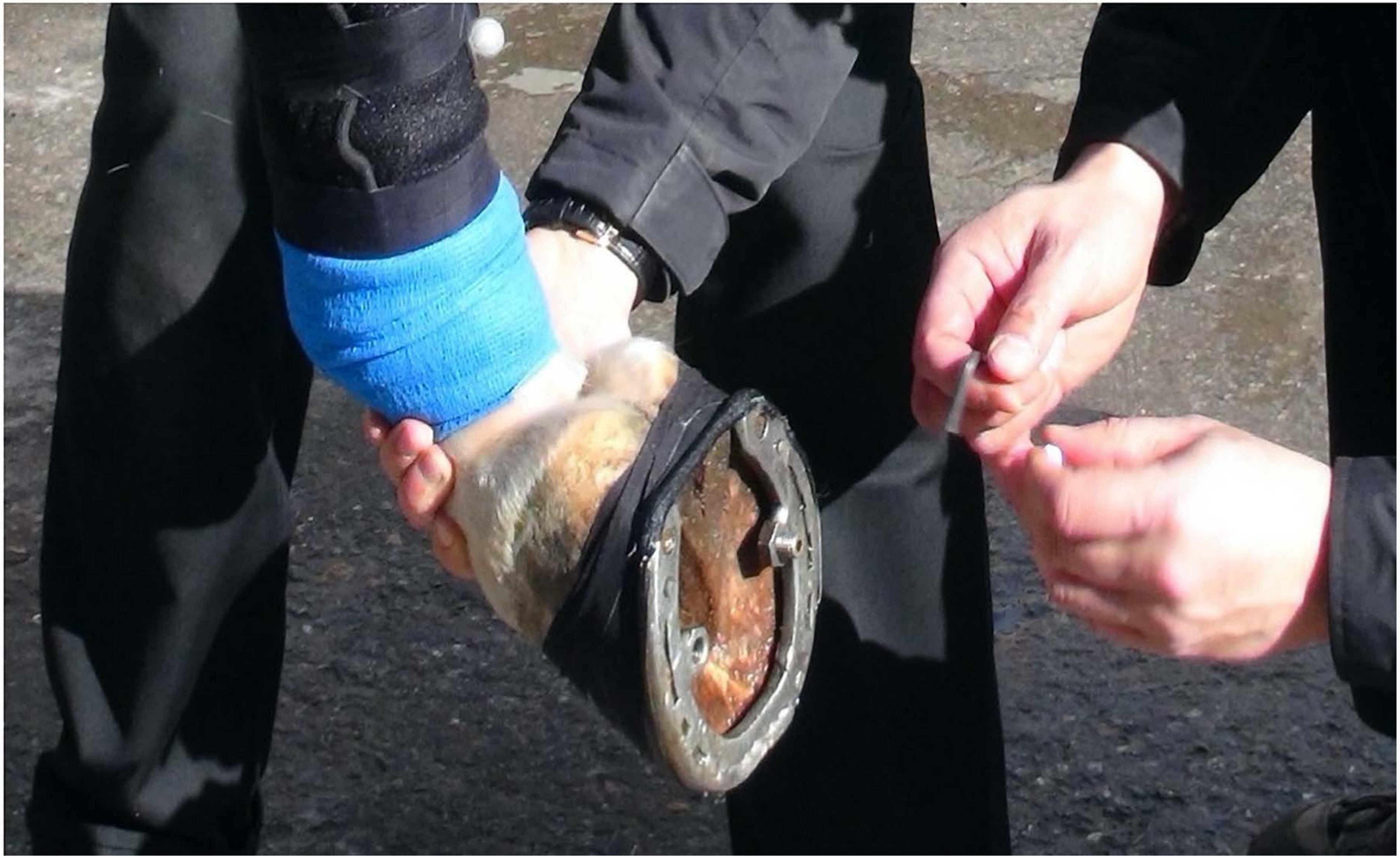

Supplement: Supplementary file 1 — Fig S1 [file EVJ-53-600-s001.pdf]
