# Supplementary material for: Adaptation strategies of horses with induced forelimb lameness walking on a treadmill
Source: Equine Vet J. 2020 Sep 24;53(3):600–11. doi: 10.1111/evj.13344 (PMC8048804; doi:10.1111/evj.13344)

Equine Veterinary Journal

Figure S2: One of the study subjects, equipped with 52 reflective markers at trot. The markers used for the analysis were: poll, withers (T5-6), dorsal spinal process of L3, tuber sacrale, dorso-cranial aspect of the left/right tuber coxae, trochanter major of the femur, left/right lateral aspect of the tarsus, sternum, left/right tuber spina scapulae, left/right lateral aspect of the carpus, left/right-front/hind lateral aspect of the fetlock, left/right-front/hind centre of rotation of the coffin joint.

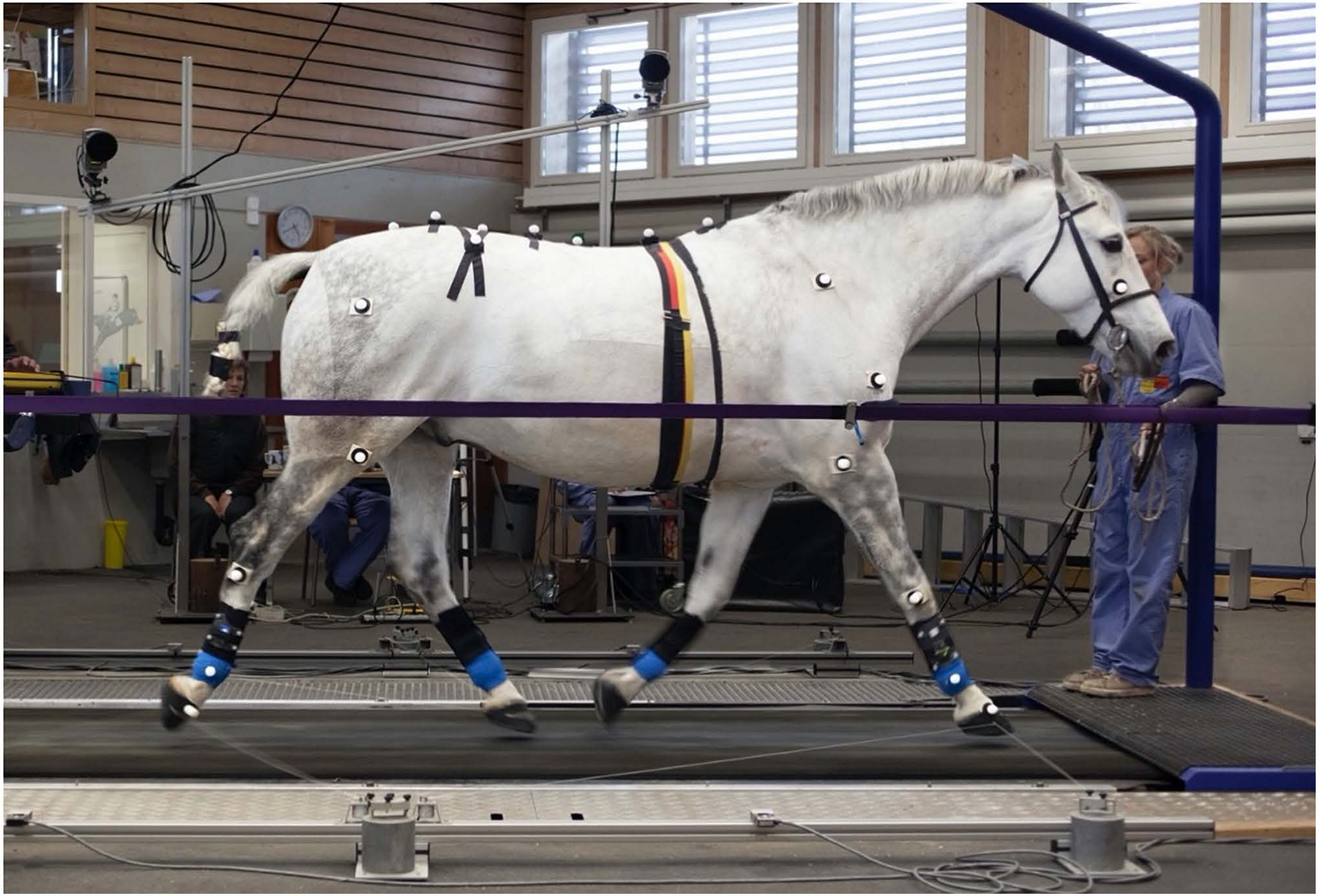

Supplement: Supplementary file 2 — Fig S2 [file EVJ-53-600-s004.pdf]
