# Supplementary material for: Adaptation strategies of horses with induced forelimb lameness walking on a treadmill
Source: Equine Vet J. 2020 Sep 24;53(3):600–11. doi: 10.1111/evj.13344 (PMC8048804; doi:10.1111/evj.13344)
Supplement: Supplementary file 3 — Table S1 [file EVJ-53-600-s002.pdf]

Equine Veterinary Journal BEVA

Table S1: Detailed description of the temporal, kinetic and kinematic parameters used in this study.

Abbreviation	Name	Description	Units	Calculation/Comment
Kinetics				
Iz	Vertical impulse	Vertical impulse; bwt normalised	Ns/kg	Integral of force over stance duration
Fz_{peak1} (walk) / Fz_{peak}(trot)	(First) vertical force peak	First vertical force peak; bwt normalised	N/kg	
Fz_{dip}	Vertical force dip	Vertical force dip (between force peak 1 and 2); bwt normalised	N/kg	walk
Fz_{peak2}	Second vertical force peak	Second vertical force peak; bwt normalised	N/kg	walk
ΔFz_{load}	Maximum loading rate	Maximum loading rate during the first half of stance	(N/kg)/s	First derivative of the force over stance duration
ΔFz_{unload}	Maximum unloading rate	Maximum unloading rate during the second half of stance	(N/kg)/s	First derivative of the force over stance duration
Time				
TFzpeak1 (walk) / TFzpeak (trot)	Time of first vertical force peak	Time (% of stance duration) to first vertical force peak	%	walk, trot
TFzdip	Time of vertical force dip	Time (% of stance duration) to vertical force dip	%	walk
TFzpeak2	Time of second vertical force peak	Time (% of stance duration) to second vertical force peak	%	walk
SD	Stride duration		s	Time between two consecutive hoof-on moments of the same limb
StD	Stance duration		s	Time between hoof-on and hoof-off moments of the same limb
StpDd LF>RH	Diagonal step duration	Time between hoof initial ground contact of lame front limb and diagonal hindlimb (LF>RH)	s	walk
StpDd RF>LH		Time between hoof initial ground contact of conteralateral front limb and ipsilateral hindlimb (RF>LH)	s	walk
StpDi RH>RF	Ipsilateral step duration	Time between hoof initial ground contact of diagonal hindlimb and contralateral front limb (RH>RF)	s	walk
StpDi LH>LF		Time between hoof initial ground contact of ipsilateral hindlimb and lame front limb (LH>LF)	s	walk
StpDc LF>RF	Contralateral step duration, forelimbs	Time between hoof initial ground contact of lame and contralateral front limb (LF>RF)	s	trot
StpDc RF>LF		Time between hoof initial ground contact of conteralateral and lame front limb (RF>LF)	s	trot
StpDc	Contralateral step duration, hindlimbs	Time between hoof initial ground contact of ipsilateral and diagonal hindlimb	s	trot
StpDc		Time between hoof initial ground contact of diagonal and ipsilateral hindlimb	s	trot
OD3_dRiL	Overlap duration of tripedal support	Two forelimbs and one hind (diagonal right and ipsilateral left)	s	walk
OD2_iL	Overlap duration of bipedal support	One forelimb and one hind (ipsilateral left)	s	walk
OD3_iLdL	Overlap duration of tripedal support	One forelimb and two hind (ipsilateral left and diagonal left)	s	walk
OD2_dL	Overlap duration of bipedal support	One forelimb and one hind (diagonal left)	s	walk
OD3_dLiR	Overlap duration of tripedal support	Two forelimbs and one hind (diagonal left and ipsilateral right)	s	walk
OD2_iR	Overlap duration of bipedal support	One forelimb and one hind (ipsilateral right)	s	walk
OD3_iRdR	Overlap duration of tripedal support	One forelimb and two hind (ipsilateral right and diagonal right)	s	walk
OD2_dR	Overlap duration of bipedal support	One forelimb and one hind (diagonal right)	s	walk
TAP	Time of advanced placement	Time dissociation between diagonal limbs at initial ground contact; positive if hindlimb preced the forelimb (dL, dR)	s	trot
Kinematics - limbs				
StL		Stance length	m	Stance duration times treadmill belt speed
Ret_{max}		Maximum limb retraction angle	deg	Forelimbs (Line between tuber spinae scapulae and fetlock); Hindlimbs (Line between hip and fetlock)
$Prot_{max}$		Maximum limb protraction angle	deg	Forelimbs (Line between tuber spinae scapulae and fetlock); Hindlimbs (Line between hip and fetlock)
$Prot_{speed}$		Maximum limb protraction speed	m/s	
$Prot_{height}$		Maximum limb protraction height, relative to treadmill	mm	
$A_{fetlock}$		Fetlock maximum hyperextension during stance phase	deg	
Kinematics - upper body				
MinDiff		Difference between left and right stride half-cycle in minimum vertical position *	mm	
MaxDiff		Difference between left and right stride half-cycle in maximum vertical position *	mm	
RUD		Difference between left and right stride half-cycle upwards vertical displacement *	mm	
RDD		Difference between left and right stride half-cycle downwards vertical displacement *	mm	
COM		Body center of mass	mm	3D midpoint between sternum and the dorsal spinal process of the lumbar vertebra L3.

* value of 0 indicates perfect symmetry; increasing values indicate an increased asymmetry.Positive values indicate an asymmetry congruent with a right limb lameness
