# Supplementary material for: Adaptation strategies of horses with induced forelimb lameness walking on a treadmill
Source: Equine Vet J. 2020 Sep 24;53(3):600–11. doi: 10.1111/evj.13344 (PMC8048804; doi:10.1111/evj.13344)
Supplement: Supplementary file 4 — Table S2 [file EVJ-53-600-s003.pdf]

Equine Veterinary Journal BEVA

Table S2: Differences in temporal and kinetic variables between baseline and induction conditions from model estimates. Values associated with the lame limb are highlighted in blue and significant differences are highlighted in green. Left fore (LF) is the lame limb. Please refer to Table S1 for variable names. SE: Standard error.

			Walk							Trot						
			Baseline		Lameness induction		Induction - Baseline			Baseline		Lameness induction		Induction - Baseline		
Variable	Units	Limb	Estimate	SE	Estimate	SE	Difference	% difference	p-value	Estimate	SE	Estimate	SE	Difference	% diffeence	p-value
Iz	Ns/kg	LF	3.28	0.06	3.11	0.06	-0.17	-5.14	<0.001	2.02	0.03	1.73	0.03	-0.29	-14.32	<0.001
		RF	3.28	0.08	3.31	0.08	0.03	1.01	0.04	1.98	0.04	2.11	0.04	0.13	6.56	<0.001
		LH	2.32	0.04	2.29	0.04	-0.03	-1.35	<0.001	1.57	0.03	1.49	0.03	-0.08	-5.26	<0.001
		RH	2.31	0.05	2.32	0.05	0.01	0.50	>0.05	1.61	0.04	1.61	0.04	0.01	0.39	>0.05
Fz_{peak1}, Fz_{peak}	N/kg	LF	5.66	0.08	5.55	0.08	-0.11	-1.97	<0.001	11.64	0.16	9.58	0.16	-2.06	-17.69	<0.001
		RF	5.58	0.07	5.71	0.07	0.13	2.33	<0.001	11.47	0.12	11.68	0.12	0.21	1.82	<0.001
		LH	4.80	0.12	4.82	0.12	0.02	0.48	>0.05	9.70	0.13	9.40	0.13	-0.29	-3.04	<0.001
		RH	4.67	0.10	4.90	0.10	0.22	4.77	<0.001	9.92	0.21	10.02	0.21	0.10	1.00	<0.001
Fz_{dip}		LF	5.22	0.09	5.23	0.09	0.00	0.10	>0.05							
		RF	5.18	0.13	5.27	0.13	0.09	1.76	<0.001							
		LH	2.78	0.05	2.83	0.05	0.05	1.89	0.01							
		RH	2.93	0.07	2.93	0.07	0.00	0.07	>0.05							
Fz_{peak2}		LF	7.00	0.12	6.58	0.12	-0.43	-6.08	<0.001							
		RF	7.06	0.10	7.19	0.10	0.13	1.89	<0.001							
		LH	4.37	0.05	4.35	0.05	-0.02	-0.49	>0.05							
		RH	4.25	0.06	4.15	0.06	-0.10	-2.24	<0.001							
ΔFz_{load}	(N/kg)/s	LF	0.146	0.008	0.145	0.008	-0.001	-0.815	>0.05	0.411	0.023	0.384	0.023	-0.027	-6.56	<0.001
		RF	0.135	0.011	0.157	0.011	0.022	16.544	<0.001	0.383	0.019	0.403	0.019	0.020	5.33	<0.001
		LH	0.125	0.004	0.129	0.004	0.004	2.887	>0.05	0.263	0.011	0.247	0.011	-0.016	-6.05	<0.001
		RH	0.125	0.003	0.130	0.003	0.004	3.530	<0.001	0.276	0.010	0.281	0.010	0.006	2.00	<0.001
ΔFz_{unload}	(N/kg)/s	LF	-0.106	0.008	-0.110	0.008	-0.004	3.851	>0.05	-0.326	0.012	-0.249	0.012	0.077	-23.75	<0.001
		RF	-0.106	0.005	-0.122	0.005	-0.016	15.031	<0.001	-0.312	0.011	-0.313	0.011	-0.001	0.22	>0.05
		LH	-0.091	0.004	-0.090	0.004	0.001	-0.638	>0.05	-0.262	0.005	-0.251	0.005	0.012	-4.42	<0.001
		RH	-0.092	0.005	-0.093	0.005	0.000	0.390	>0.05	-0.271	0.008	-0.266	0.008	0.005	-1.81	<0.001
SD	sec		1.125	0.02	1.102	0.02	-0.023	-2.047	<0.001	0.714	0.01	0.689	0.01	-0.025	-3.449	<0.001
StD_{abs}		LF	0.705	0.01	0.693	0.014	-0.012	-1.691	<0.001	0.287	0.00	0.293	0.005	0.006	1.922	<0.001
		RF	0.703	0.02	0.692	0.015	-0.011	-1.605	<0.001	0.288	0.00	0.294	0.005	0.006	2.054	<0.001
		LH	0.701	0.01	0.692	0.012	-0.009	-1.282	<0.001	0.270	0.00	0.268	0.002	-0.002	-0.843	<0.001
		RH	0.704	0.01	0.696	0.012	-0.009	-1.214	<0.001	0.269	0.00	0.268	0.002	-0.001	-0.379	>0.05
StD_{rel}	%	LF	0.626	0.003	0.629	0.003	0.003	0.44	0.0265	0.403	0.004	0.425	0.004	0.022	5.51	<0.001
		RF	0.625	0.004	0.628	0.004	0.003	0.48	>0.05	0.403	0.004	0.426	0.004	0.023	5.68	<0.001
		LH	0.624	0.003	0.632	0.003	0.007	1.16	<0.001	0.379	0.005	0.389	0.005	0.010	2.70	<0.001
		RH	0.624	0.002	0.628	0.002	0.004	0.57	0.0203	0.378	0.005	0.390	0.005	0.012	3.21	<0.001
TFz_{peak1}, TFz_{peak}	% StD	LF	29.05	0.84	31.49	0.85	2.44	8.41	<0.001	49.47	0.45	50.92	0.45	1.45	2.93	<0.001
		RF	29.55	1.07	29.98	1.07	0.43	1.45	>0.05	49.56	0.56	49.84	0.56	0.29	0.58	0.02
		LH	18.52	0.41	19.52	0.41	1.01	5.44	<0.001	50.90	0.46	50.00	0.45	-0.90	-1.76	<0.001
		RH	18.73	0.68	20.00	0.68	1.26	6.73	<0.001	50.81	0.39	49.82	0.39	-0.99	-1.95	<0.001
TFz_{dip}		LF	42.46	0.80	43.34	0.81	0.88	2.08	>0.05							
		RF	42.10	0.52	43.11	0.52	1.00	2.38	<0.001							
		LH	52.24	0.73	52.84	0.73	0.60	1.14	>0.05							
		RH	52.68	1.14	54.81	1.14	2.13	4.05	<0.001							
TFz_{peak2}		LF	66.64	1.04	66.95	1.04	0.31	0.47	>0.05							
		RF	66.53	0.83	67.78	0.83	1.25	1.89	<0.001							
		LH	76.61	0.44	77.44	0.44	0.83	1.09	<0.001							
		RH	76.38	0.77	77.07	0.77	0.69	0.90	>0.05							
StpDd	% stride	LF>RH	23.90	0.82	24.17	0.82	0.28	1.16	>0.05							
		RF>LH	26.26	0.83	26.13	0.83	-0.13	-0.50	>0.05							
StpDi		RH>RF	23.71	0.85	24.28	0.85	0.57	2.42	<0.001							
		LH>LF	26.14	0.85	25.42	0.85	-0.72	-2.76	<0.001							
StpDc		LF>RF								50.24	0.28	48.91	0.28	-1.33	-2.64	<0.001
		RF>LF								49.77	0.28	51.08	0.28	1.31	2.64	<0.001
		LH>RH								49.79	0.27	50.52	0.27	0.73	1.46	<0.001
		RH>LH								50.19	0.26	49.50	0.26	-0.69	-1.38	<0.001
OD3_dRiL			12.45	0.28	12.66	0.28	0.21	1.69	>0.05							
OD2_iL			13.18	0.74	13.06	0.74	-0.13	-0.96	>0.05							
OD3_iLdL			12.42	0.19	13.21	0.19	0.79	6.37	<0.001							
OD2_dL			11.82	0.79	10.90	0.79	-0.93	-7.84	<0.001							
OD3_dLiR			12.63	0.45	13.02	0.45	0.39	3.07	<0.001							
OD2_iR			13.07	0.73	13.34	0.73	0.27	2.04	>0.05							
OD3_iRdR			12.43	0.23	12.64	0.23	0.22	1.77	0.081							
OD2_dR			12.01	0.82	11.17	0.82	-0.84	-6.97	<0.001							
TAP		dL								0.006	0.003	-0.006	0.003	-0.012		<0.001
		dR								0.006	0.003	-0.010	0.003	-0.016		<0.001
