# Supplementary material for: Adaptation strategies of horses with induced forelimb lameness walking on a treadmill
Source: Equine Vet J. 2020 Sep 24;53(3):600–11. doi: 10.1111/evj.13344 (PMC8048804; doi:10.1111/evj.13344)
Supplement: Supplementary file 5 — Table S3 [file EVJ-53-600-s006.pdf]

Equine Veterinary Journal BEVA

Table S3: Differences in the kinematic variables between baseline and induction conditions from model estimates. Values associated with the lame limb are highlighted in blue and significant differences highlighted in green. Left fore (LF) is the lame limb. Please refer to Table S1 for variable names.

			Walk							Trot						
			Baseline		Lameness induction		Induction - Baseline			Baseline		Lameness induction		Induction - Baseline		
	Units	Location	Estimate	SE	Estimate	SE	Difference	% difference	p-value	Estimate	SE	Estimate	SE	Difference	% difference	p-value
MinDiff	mm	Head	0.6	6.4	-35.2	6.4	-35.8		<0.001	1.4	5.2	-67.1	5.2	-68.5		<0.001
MaxDiff			3.1	5.7	8.0	5.7	4.9		>0.05	5.1	3.3	-35.6	3.2	-40.7		<0.001
RUD			-2.5	10.7	-43.1	10.8	-40.6		<0.001	6.3	6.7	-102.8	6.7	-109.1		<0.001
RDD			3.9	5.7	-27.4	5.8	-31.3		<0.001	-3.7	5.5	-31.5	5.5	-27.8		<0.001
ROM_z (dorso-ventral)			120.6	5.5	128.7	5.5	8.1	6.70	0.003	80.0	5.9	120.4	5.9	40.4	50.5	<0.001
MinDiff		Withers	2.3	1.1	-5.2	1.1	-7.4		<0.001	-0.5	1.7	-16.3	1.7	-15.8		<0.001
MaxDiff			1.0	0.8	0.2	0.8	-0.8		>0.05	-3.8	2.0	-2.8	1.9	0.9		>0.05
RUD			-3.3	1.7	4.9	1.7	8.2		<0.001	-4.2	3.2	-19.2	3.2	-14.9		<0.001
RDD			1.3	1.1	-5.3	1.1	-6.6		<0.001	3.3	1.7	-13.5	1.7	-16.8		<0.001
ROMz (dorso-ventral)			35.9	2.8	36.6	2.8	0.7	1.9	>0.05	80.3	2.9	76.7	2.9	-3.6	-4.5	<0.001
$Prot_{max}$	deg	LF	20.1	1.0	20.0	1.0	-0.1	-0.4	>0.05	18.6	0.9	18.9	0.9	0.3	1.6	<0.001
		RF	19.1	0.9	18.5	0.9	-0.5	-2.8	<0.001	18.0	0.7	18.2	0.7	0.1	0.6	>0.05
		LH	19.7	0.8	19.1	0.8	-0.7	-3.3	<0.001	17.6	0.7	18.1	0.7	0.5	2.8	<0.001
		RH	19.5	0.8	19.1	0.8	-0.3	-1.7	<0.001	17.7	0.8	17.8	0.8	0.1	0.5	>0.05
Ret_{max}		LF	23.1	0.5	22.5	0.5	-0.6	-2.6	<0.001	22.6	0.5	22.0	0.5	-0.6	-2.8	<0.001
		RF	22.9	0.6	22.2	0.6	-0.8	-3.3	<0.001	22.5	0.7	22.6	0.7	0.1	0.5	>0.05
		LH	28.2	0.6	27.9	0.6	-0.4	-1.3	>0.05	22.7	0.4	22.9	0.4	0.2	0.9	>0.05
		RH	28.3	0.8	27.6	0.8	-0.7	-2.5	<0.001	23.0	0.8	21.7	0.8	-1.3	-5.5	<0.001
$A_{fetlock}$		LF	-53.8	1.8	-52.0	1.8	1.8	-3.3	<0.001	-66.4	1.8	-60.4	1.8	6.0	-9.0	<0.001
		RF	-52.0	2.3	-52.8	2.3	-0.8	1.6	<0.001	-65.4	2.6	-66.0	2.6	-0.6	0.9	0.004
		LH	-45.1	1.6	-46.4	1.6	-1.4	3.0	<0.001	-60.6	2.3	-60.9	2.3	-0.4	0.6	<0.001
		RH	-42.7	2.1	-44.3	2.1	-1.6	3.7	<0.001	-58.8	2.6	-60.2	2.6	-1.4	2.4	<0.001
$Prot_{height}$	mm	LF	101.7	1.7	102.2	1.7	0.5	0.5	>0.05	140.1	3.2	116.5	3.2	-23.5	-16.8	<0.001
		RF	101.9	2.8	104.4	2.8	2.5	2.5	<0.001	138.1	2.9	141.9	2.9	3.7	2.7	<0.001
		LH	137.4	6.9	135.7	6.9	-1.8	-1.3	>0.05	148.3	7.6	155.5	7.6	7.2	4.9	<0.001
		RH	135.4	7.2	137.3	7.2	1.9	1.4	>0.05	152.5	8.0	163.5	8.0	11.0	7.2	<0.001
$Prot_{speed}$	m/s	LF	3.01	0.07	2.87	0.07	-0.14	-4.6	<0.001	6.28	0.07	6.11	0.07	-0.17	-2.7	<0.001
		RF	3.03	0.08	3.01	0.08	-0.01	-0.4	>0.05	6.28	0.06	6.36	0.06	0.08	1.3	<0.001
		LH	3.18	0.09	3.25	0.09	0.08	2.4	<0.001	6.75	0.11	6.73	0.11	-0.03	-0.4	>0.05
		RH	3.15	0.08	3.23	0.08	0.09	2.7	<0.001	6.77	0.14	6.82	0.14	0.04	0.6	>0.05
StL	m	LF	1.17	0.02	1.15	0.02	-0.02	-2.0	<0.001	1.10	0.02	1.11	0.02	0.02	1.4	<0.001
		RF	1.17	0.02	1.15	0.02	-0.02	-1.8	<0.001	1.09	0.01	1.12	0.01	0.02	2.0	<0.001
		LH	1.13	0.02	1.11	0.02	-0.03	-2.2	<0.001	0.99	0.01	0.97	0.01	-0.02	-1.6	<0.001
		RH	1.13	0.02	1.11	0.02	-0.02	-1.7	<0.001	0.99	0.01	0.99	0.01	0.00	0.3	>0.05
ROM_X (horizontal fore-aft)	mm	COM	15.6	1.6	18.2	1.6	2.6	16.4	<0.001	24.3	2.0	23.7	2.0	-0.6	-2.6	>0.05
ROM_Y (horizontal lateral)			83.2	4.7	76.2	4.7	-7.0	-8.4	<0.001	31.2	2.0	31.2	2.0	0.0	-0.1	>0.05
ROM_Z (vertical)			34.7	2.4	37.3	2.4	2.6	7.6	<0.001	91.9	5.8	83.9	5.8	-8.0	-8.7	<0.001
